# Mechanical and Magnetic Properties of Double Layered Nanostructures of Tin and Zirconium Oxides Grown by Atomic Layer Deposition

**DOI:** 10.3390/nano11071633

**Published:** 2021-06-22

**Authors:** Aile Tamm, Helle-Mai Piirsoo, Taivo Jõgiaas, Aivar Tarre, Joosep Link, Raivo Stern, Kaupo Kukli

**Affiliations:** 1 Institute of Physics, University of Tartu, W. Ostwaldi 1, 50411 Tartu, Estonia; helle-mai.piirsoo@ut.ee (H.-M.P.); taivo.jogiaas@ut.ee (T.J.); aivar.tarre@ut.ee (A.T.); kaupo.kukli@ut.ee (K.K.); 2 Laboratory of Chemical Physics, National Institute of Chemical Physics and Biophysics, Akadeemia tee 23, 12618 Tallinn, Estonia; joosep.link@kbfi.ee (J.L.); raivo.stern@kbfi.ee (R.S.)

**Keywords:** atomic layer deposition, nanostructures, magnetization, nanoindentation, tin dioxide, zirconium dioxide

## Abstract

Double layered stacks of ZrO_2_ and SnO_2_ films, aiming at the synthesis of thin magnetic and elastic material layers, were grown by atomic layer deposition to thicknesses in the range of 20–25 nm at 300 °C from ZrCl_4_, SnI_4_, H_2_O, and O_3_ as precursors. The as-deposited nanostructures consisted of a metastable tetragonal polymorph of ZrO_2_, and a stable tetragonal phase of SnO_2_, with complementary minor reflections from the orthorhombic polymorph of SnO_2_. The hardness and elastic modulus of the stacks depended on the order of the constituent oxide films, reaching 15 and 171 GPa, respectively, in the case of top SnO_2_ layers. Nonlinear saturative magnetization could be induced in the stacks with coercive fields up to 130 Oe.

## 1. Introduction

Magnetic thin solid films can be of interest as functional materials tailoring different physical properties such as ferromagnetism as well as mechanical elasticity [1,2]. The ability to adjust the magnetic properties of a system without drastically changing other physical properties, such as electrical, mechanical, or thermal properties, could be beneficial for applications in magnetic micro- and nanoelectromechanical systems [3]. In addition, electrodeposited soft magnetic materials are used as writing heads for hard disks, where the main requirements include corrosion resistance, low stress, and high thermal stability [4]. One might seek materials systems with high corrosion resistance, mechanical hardness, and elasticity, at the same time using materials of higher transparency. Some alloys of metals can be regarded as materials simultaneously exhibiting magnetism and elasticity. Complementarily, optical transparency may herewith be achieved if the films would possess nanocrystalline nature [1]. Magnetization performance may in such materials become directly affected by local changes in mechanical strain and stress [5,6,7]. In this regard, nanoindentation studies on hardness and elasticity are relevant, especially when considering the materials for applications like magnetic recording, where mechanical properties of the recording medium and reading head surface become important. Magnetic thin films are often based on metallic thin films like Fe, Ta, Co, or Cr [8] and are less based on oxides. On some occasions, the thin film thickness is kept relatively high at 600 nm [9] to avoid any influence from deposition substrate on thin-film properties during nanomechanical characterization. On the other hand, some publications report the magnetic and mechanical properties of thin films with thicknesses around 70 nm [8]. In the latter article, the authors admit that to measure directly only the thin film properties, they should be able to measure properties at a nanoindenter tip displacement of 7 nm or below to avoid substrate influence and this task probably cannot be fulfilled. Due to developments in measurement techniques and technology in recent years, the possibility to mechanically characterize ultra-thin films on a substrate has almost become a viable option [1,2,3,5,7].

Transparent conducting magnetic semiconductors, e.g., bismuth-doped ZnO [10] can be considered as alternatives to metal alloys. Further, accommodation of wide band-gap transition metal oxides, i.e., insulators, instead of metals and semiconductors might more feasibly provide high transparency in the visual range, whereby the nonlinear magnetization in such oxides could be achieved by the engineering of their dimensions and, concurrently, their phase composition. Synthesis processes of metal oxides might also be considered as relatively inexpensive and more robust, compared to those of heavy and noble metals, in addition to their compatibility to a large area and low-temperature deposition techniques, such as atomic layer deposition (ALD). One might expect the formation and presence of metastable polymorphs, possessing useful physical properties, in nanocomposites of different metal oxides, such as, for example, SnO_2_ and ZrO_2_. SnO_2_ is a transparent, wide-band-gap oxide semiconductor that is applied widely in many fields of oxide electronics [11,12], owing to its good optical and electrical properties and excellent chemical and thermal stability [13].

Mixtures of SnO_2_ and ZrO_2_ in thin-film form, obtained by pulsed laser deposition, have performed as transparent conductors [14]. Optically fully transparent stacks of sol-gel synthesized ZrO_2_ and SnO_2_ films have been studied as gate dielectrics on channel layers, respectively, in transistor devices [15]. Tin-rich transparent mixtures of SnO_2_ and ZrO_2_, synthesized by chemical solution deposition, have demonstrated nonlinear saturative magnetization, increasing with the content of zirconium, under an external magnetic field [16].

One could propose deposition and engineering of nanocomposite material layers in stacks, instead of mixtures, in order to provide constituent functional metal oxides of distinct composition and controlled structure. In this way, the formation (ordering) of possible phases would more likely be defined either by the influence of the structure of substrates or the thickness of the films limiting the crystal growth, instead of the cation ratio in mixtures.

The present study has been devoted to the synthesis of ZrO_2_ and SnO_2_ thin films in stacked layers by ALD. The precursor chemistry was based on ALD processes using ZrCl_4_ and H_2_O [17], and SnI_4_ and O_3_ [18] as precursors. The investigations compared the evaluation of the crystallographic phase composition, vibrating sample magnetometry, and instrumented nanoindentation.

## 2. Materials and Methods

The films were grown in a flow-type in-house-built hot-wall ALD reactor [19] from tin(IV)iodide (SnI_4_, 95%, Sigma Aldrich, Darmstadt, Germany) and zirconium(IV)chloride (ZrCl_4_, >99.95%, Strem, Newburyport, MA, USA) as metal precursors, whereby O_3_ and H_2_O as oxygen precursors were used together with SnI_4_ and ZrCl_4_, respectively. N_2_ (99.999%) was used as the carrier and purge gas. SnI_4_ was evaporated at 104 ± 2 °C and ZrCl_4_ at 156 ± 2 °C from open boats inside the reactor and transported to the substrates by the carrier gas flow. During the deposition, the chamber pressure remained in the range of 210–230 Pa. The precursor pulse durations and purge lengths for SnO_2_ were 5-2-5-5 s and for ZrO_2_ was 4-3-2-5 s, denoting the sequences of metal precursor pulse—N_2_ purge—H_2_O/O_3_ pulse—N_2_ purge. SnO_2_-ZrO_2_ layered nanostructures were deposited in the following manner: 60 × SnO_2_ (60 ALD growth cycles of SnI_4_ + O_3_) + 150 × ZrO_2_ (150 ALD growth cycles of ZrCl_4_ + H_2_O). Alternatively, the order of layers was reversed from SnO_2_—ZrO_2_ to ZrO_2_—SnO_2_. The reference samples of 120 cycles SnO_2_ and 300 cycles ZrO_2_ were also prepared, grown to the comparable thicknesses. All the films were deposited on silicon (100) substrates at 300 °C. Prior to the deposition, the substrates were degreased in the hot mixture of H_2_SO_4_-H_2_O_2_, followed by etching in a HF solution to remove the native oxide, and rinsed in deionized water.

The crystal structure was evaluated by grazing incidence X-ray diffractometry (GIXRD), by using a SmartLab (Rigaku, Tokyo, Japan) tool with the incidence angle of 0.42 deg and the CuKα radiation, which corresponds to an X-ray wavelength of 0.154063 nm. The morphology of the layered structures on Si substrate was investigated by scanning electron microscopy (SEM) by using Helios Nanolab 600 DualBeam (FIB) microscope.

**Figure 1:**
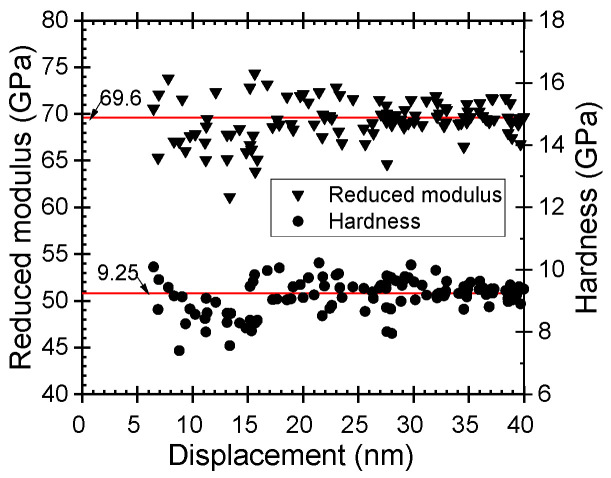
Nanoindentation tip calibration results for reduced elastic modulus and hardness against indentation depth. Solid horizontal lines indicate the reference values of 69.6 GPa and 9.25 GPa, for the modulus and hardness, respectively, characteristic of fused quartz.

The hardness and Young’s modulus of the films were determined using an instrumented nanoindentation of Bruker’s TriboIndenter TI980 (Eden Prairie, MN, USA). The Berkovich tip was calibrated prior to the measurements on a fused quart glass reference sample with a hardness of 9.25 GPa and a reduced modulus of 69.6 GPa. The standard deviation for hardness and modulus were 0.46 and 1.85, respectively, in the displacement range from 5 to 50 nm. Indentation was carried out in both quasi and dynamic (continuous stiffness measurement) modes with forces varying from 50 to 250 µN. In addition, the indents were characterized by the scanning probe microscopy (SPM) method applied by the same TriboIndenter and refined with open software Gwyddion 2.56. Displacement regions where the measured values matched with the properties of the fused quartz glass were calibrated (Figure 1) and could, thereafter, be regarded as the trustworthy indentation range.

Selected films were subjected to magnetic measurements performed by using the P525 Vibrating Sample Magnetometer (VSM) option of the Physical Property Measurement System (PPMS) 14T (Quantum Design). Rectangular samples (about 5 × 5 mm^2^) were fixed with GE 7031 varnish to the commercial quartz sample holders (Quantum Design). Hysteresis measurements were carried out at room temperature (300 K) by scanning the magnetic field from −40,000 to +40,000 Oe (from −3183.098 to 3183.098 kA/m) parallel to the film surface. The diamagnetic signal, arising from the silicon substrate, was subtracted from the general magnetization curve for all samples in which the ferromagnetic-like response was detected.

## 3. Film Structure and Morphology

The films were grown to thicknesses not exceeding 25 nm. Such rather thin and expectedly nanocrystalline stacked layers were built as those being of interest in terms of magnetic nanostructures. At the same time, the low thickness of the stacked layers was also expected to create challenges in the evaluation of mechanical properties using instrumented nanoindentation. The films were grown on silicon as well as on metal oxide substrates. Average growth rates for the SnO_2_ and ZrO_2_ films at 300 °C were 0.21 and 0.07 nm/cycle, respectively, as estimated on the basis of ex-situ XRR measurements. The thicknesses and densities of the layered nanostructures are presented in Table 1.

**Figure 2:**
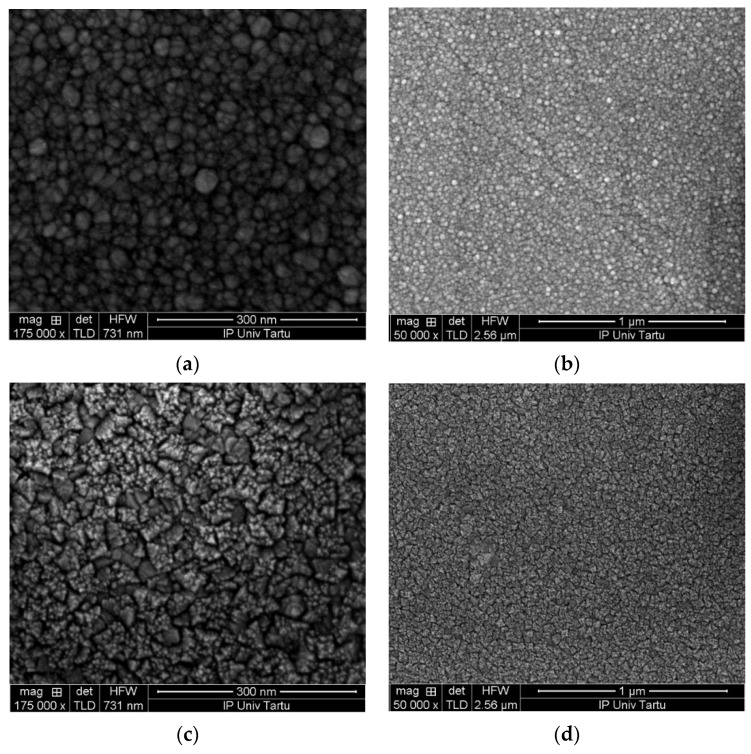
Scanning electron microscope bird-eye images of top SnO_2_ (**a**,**b**) and ZrO_2_ (**c**,**d**) layers in stacked nanostructures.

Scanning electron microscope images (Figure 2) demonstrated that the surfaces of both tin and zirconium oxide films were quite uniformly covered by grain-like features. Those features were slightly different, i.e., in the case of the tin oxide, the grains appeared round-like (Figure 2a,b) whereas, in the case of the zirconium oxide, the features visible on the surface were formed more like triangles (Figure 2c,d). These surface features could be connected to crystallization already after the primary visual inspection. These features indicated that the deposition temperature of 300 °C was high enough to initiate crystal growth in both oxide layers grown to thicknesses as low as 10–15 nm.

**Figure 3:**
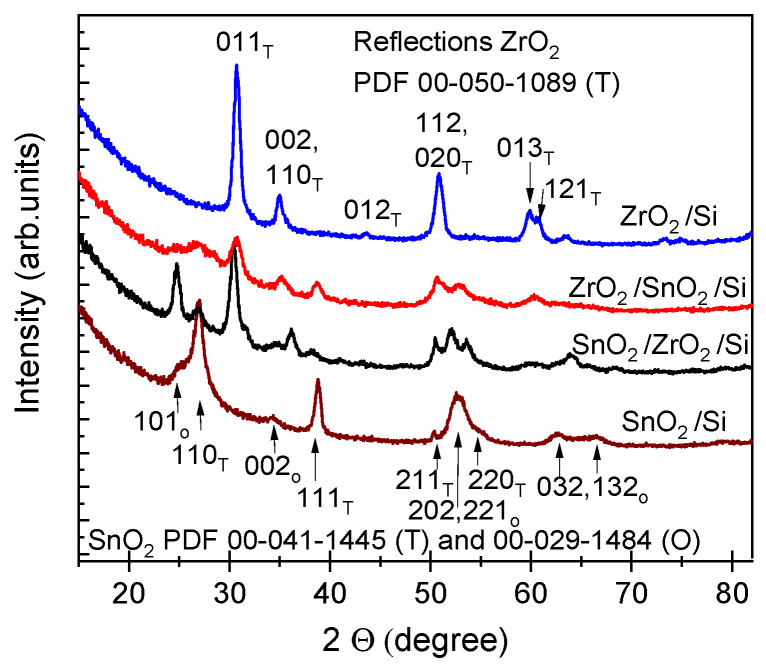
X-ray diffraction patterns from the SnO_2_ and ZrO_2_ thin films and ZrO_2_-SnO_2_ stacked layered nanostructures, in the as-deposited state of thin films. Miller indexes assigned after crystallization are indicated with “T” for the tetragonal phase of ZrO_2_, “T” and “O” for the tetragonal and orthorhombic phases of the SnO_2_ accordingly.

The GIXRD patterns confirmed that all the double-layered nanostructures were indeed crystallized already in the as-deposited state. The diffractogram of the reference ZrO_2_ thin film (Figure 3, topmost pattern) revealed clear peaks at 30.7°, 34.9°, 43.6°, 50.8°, 59.7°, and 60.7°, attributable to those of 011, 002/110, 012, 112/020, 013, and 121 reflections respectively, of the tetragonal ZrO_2_ phase (PDF card 00-050-1089). The diffractogram of the reference SnO_2_ thin film (Figure 3, the lowest pattern) revealed at 26.9°, 38.8°, and 52.6/54.5°, attributable to those of 110, 111, and 211/220 reflections respectively, of the tetragonal SnO_2_ phase (PDF card 00-041-1445). Relatively weak reflections of the orthorhombic phase of SnO_2_ were also observed in the case of films containing tin oxide layer at 25.0°, 34.3°, 52.3°, 62.7°, and 66.3°, attributable to 101, 002, 202, 221, 023, and 132 reflections, orthorhombic SnO_2_ phase (PDF Card 00-029-1484). The common phase for SnO_2_, also in the thin film form, is tetragonal cassiterite, whereas the orthorhombic phase of SnO_2_ could become the stable one at high pressures and temperatures. Referring to earlier studies, pulsed laser deposition of metastable orthorhombic SnO_2_ films with improved optically transparency has been carried out on Si(100) substrates at 320 °C [20] and the stabilization of that phase has been attributed to the presence and exchange reactions of oxygen vacancies at nanocrystallite boundaries in the growing films. Epitaxial orthorhombic SnO_2_ films have been grown on stabilized cubic ZrO_2_:Y films by metal-organic chemical vapor deposition from tetraethyltin, Sn(C_2_H_5_)_4_, and O_2_ at 500 and 600 °C [21]. Considering possible intermixing of solid layers and formation of ternary compounds, orthorhombic ZrSnO_4_ phase has been observed previously in calcined nanocomposites of ZrO_2_ and SnO_2_ prepared by sol-gel technique [22], but reflections of such ternary compounds were not observed in the present study. Thus, the constituent oxides must have grown on top of each other and formed nanocrystalline stacks of two different components.

## 4. Mechanical Properties

**Figure 4:**
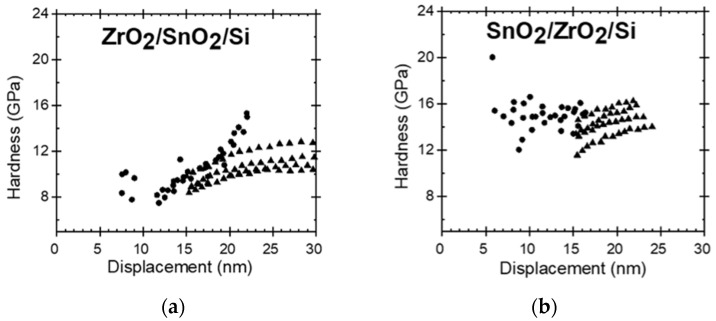
Hardness along the depth of the ZrO_2_/SnO_2_/Si (**a**) and SnO_2_/ZrO_2_/Si (**b**) stacked layered nanostructures. Results of quasi-static indentation measurements (●) and dynamic measurements (▲).

**Figure 5:**
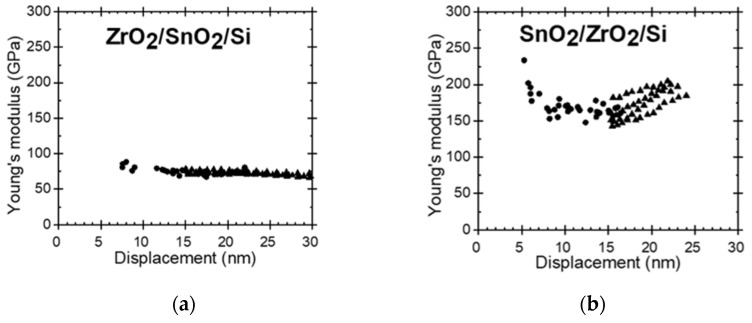
Young’s modulus along the depth of the ZrO_2_/SnO_2_/Si (**a)** and SnO_2_/ZrO_2_/Si (**b**) stacked layered nanostructures. Results of quasi-static indentation measurements (●) and dynamic measurements (▲).

The nanoindentation results are presented in Table 2, where the geometric mean values for hardness and Young’s modulus are given for each film. Thereby, the 21 nm thick ZrO_2_/Si film possessed the hardness of 11.5 GPa and elastic modulus of 96 GPa, which are slightly lower values than those measured in approximately three times thicker films deposited earlier in a similar ALD process [23,24]. The 25 nm thick SnO_2_/Si film possessed noticeably higher hardness and stiffness than that based on the ZrO_2_. For the ZrO_2_- SnO_2_ stacked nanostructures, the order of layers has influenced the mechanical properties (Figure 4). For the layers stacked with the harder and stiffer SnO_2_ on top of ZrO_2_, the hardness and Young’s modulus of the stack resembled those of the SnO_2_ film (Figure 4a). At the same time, when the SnO_2_ layer was deposited first, below the ZrO_2_, the mechanical properties of the stack started to resemble those of the ZrO_2_ film (Figure 4b). The substrate was characterized by the hardness similar to that of the softer oxide films, and the average elastic modulus remaining between those of the harder and softer oxide films (Figure 5).

The nanoindentation measurements are presented in Figure 4 and Figure 5. Neither hardness nor elastic modulus seemed to change at different depths. The likely influence of the substrate did not become evident during the indentation measurement procedures. However, considering the film thicknesses and the measured depth ranges, the results should be influenced by the substrate. The films were grown to thicknesses less than 30 nm, and at indentation depths exceeding 10% of the film thickness the effect of the substrate must become significant [25,26].

The initial work of Jönsson and Hogmark [27] states that the substrate starts to influence the coating-substrate measurement results when the indentation displacements are around 0.07–0.2 times the coating thickness. Therefore, it became evident that specific models have to be developed to find the properties of the coating alone. Over the years, several researchers have proposed different models to perform such calculations, in addition to Jöhnsson and Hogmark, for instance, Burnett and Rickerby [28], Chicot and Lesage [29], and Tuck and Korsunsky [30]. In one of the latest works, written by Puchi-Cabrera, a comprehensive comparison of named models and an additional model is proposed [31]. The problem with the calculation of the sole coating property is that the coatings are never used as a single piece of material but it covers some other material and therefore the performance is usually the combination of the two. Therefore, it is rather reasonable to create a specific coating-substrate system with emphasis on a probable application and then characterize the stack as a whole. Therefore, any attempt to calculate the sole property of the deposited thin films is omitted here.

**Figure 6:**
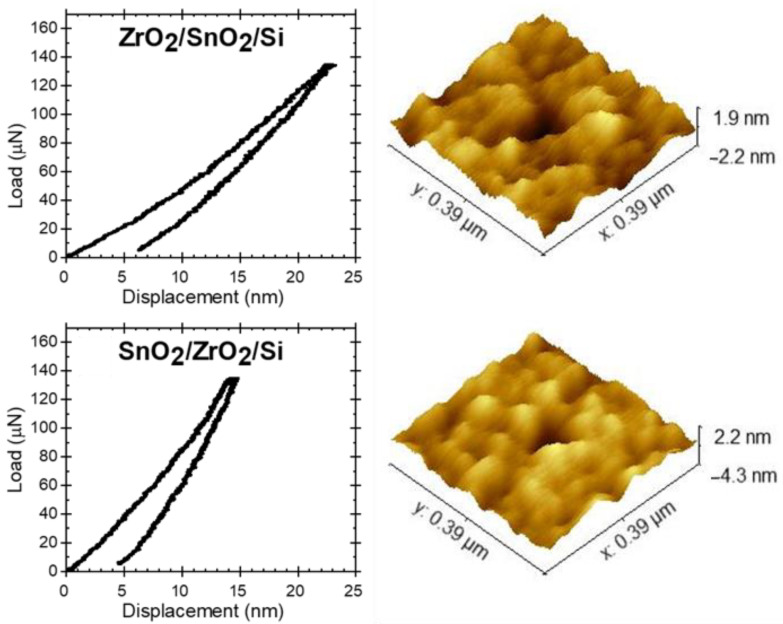
The load-displacement curves (**a**) and scanning probe microscopy images (**b**) of the ZrO_2_/SnO_2_/Si (**a**) and SnO_2_/ZrO_2_/Si (**b**) stacked layered nanostructures. The corresponding load-displacement curves produced the indents with a maximum load of 135 µN.

The results obtained in separate measurements were considerably scattered (Table 2). This can be attributed to the complications with the calibration of the indenter tips within small displacements, heterogeneity, and anisotropy of the measured materials [25], or the absence of considerable plastic deformation at such low indentation loads. Hardness can be determined when plastic deformation occurs, whereas, during the measurements in the present study, one had to apply forces weaker than those required to produce sufficient extents of plastic deformation. Figure 6 shows the SPM images of the indents and the corresponding load-displacement graphs (Figure 6a) with a maximum indentation force of 135 µN. At this indentation load (in the quasi mode) the indentation depth of the film SnO_2_ is comparable to the surface roughness in Table 1 (Figure 6b). Decreasing the indentation load diminished the discernibility of the indents further, meaning that little or no plastic deformation occurred, making the hardness, estimated at applied loads smaller than 135 µN, unreliable. The average hardness presented in Table 2 was calculated using results acquired at higher loads and from dynamic measurements.

The mechanical properties of the stacked films were influenced significantly by the order of the layers. A theoretical study by Pelegri et al. [26] on the behavior of hardness and Young modulus in hard-film-soft-substrate and soft-film-hard-substrate systems indicated tendencies somewhat similar to the results obtained in the present study. In the present study, for the nanostructures with the top ZrO_2_ (softer) layer and bottom SnO_2_ (harder) layer, the hardness was similar to that of the ZrO_2_ film. However, the Young modulus of such nanostructure was slightly lower compared to the ZrO_2_ reference film which could be due to the influence of the Si substrate that has lower stiffness than SnO_2_. Pelegri et al. [26] found that the hard-film-soft-substrate system should possess the same hardness as the hard-bulk material, yet lower Young modulus. In the present study, the SnO_2_/ZrO_2_/Si system was measured by the same hardness as SnO_2_/Si, and also similar modulus which again could be the result of the influence of the Si substrate [27,30,31].

## 5. Magnetic Properties

**Figure 7:**
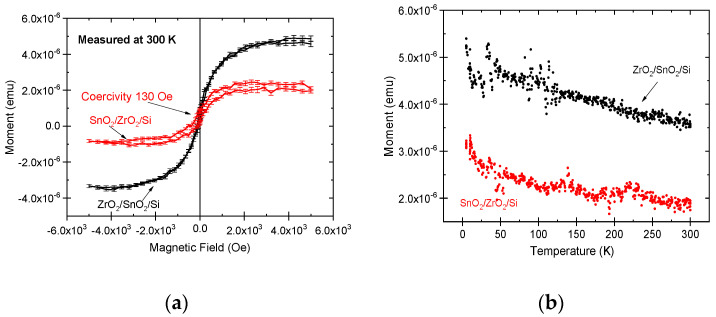
Magnetization versus external field curves measured at room temperature (**a**) and temperature dependencies of magnetization, recorded at 1 kOe (**b**), in stacked layers of SnO_2_ and ZrO_2_, as denoted by labels.

Magnetic measurements were performed for as-deposited stacked nanostructures by applying a magnetic field in the film plane. The structures demonstrated at room temperature nonlinear hysteretic magnetization, generally characteristic to ferromagnetic, ferrimagnetic, and superparamagnetic materials, at room temperature with saturation magnetization (Figure 7a). In the magnetization-field (M-H) curves, the saturation magnetization values for the SnO_2_/ZrO_2_/Si and ZrO_2_/SnO_2_/Si nanostructures reached 1.5 × 10^−4^ and 3.5 × 10^−4^ emu, respectively. Both double-layered nanostructures showed measurable coercivity, ranging approximately from 65 to 130 Oe (5.2 to 10.4 kA/m). The saturation magnetization M_s_, recorded at 1 kOe against temperature (Figure 7b), followed a linear trend, decreasing moderately with increasing temperature. Single ZrO_2_ film on Si substrate exhibited magnetization lower than 2 × 10^−6^ emu and insignificant coercivity, which is also consistent with the results of our earlier studies [32]. However, single ZrO_2_ films, grown to higher thicknesses, otherwise can exhibit considerable hysteretic magnetization, nonlinear in external fields, as has been observed in several works earlier [33,34,35], probably due to the stabilization and presence of metastable phases, rich of oxygen vacancies. The latter inevitably accompanies the nanocrystalline nature of the thin solid structures and concurrent stabilization of metastable polymorphs. The role of vacancies as likely the main factor affecting the surface energies and inducing stabilization of metastable polymorphs in nanocrystalline ZrO_2_ has earlier been studied and is a widely recognized phenomenon [36,37,38,39,40]. Magnetic properties may, in a somewhat similar manner, appear and become measurable in SnO_2_ based oxide films. The room temperature ferromagnetism has been detected in SnO_2_-based nanostructures not mixed with foreign cations, as reported in several papers [41,42,43,44].

## 6. Conclusions

SnO_2_ and ZrO_2_ stacked double-layered nanostructures were deposited by atomic layer deposition using SnI_4_, ZrCl_4_, O_3,_ and H_2_O. Layered structures consisting of chemically distinct metal oxide layers were formed at a substrate temperature of 300 °C on silicon substrates. The ZrO_2_ and SnO_2_ layers contained complementary and presumably oxygen-deficient metastable phases, as indicated by the presence of reflections from tetragonal and orthorhombic polymorphs, respectively, in their X-ray diffraction patterns.

The films were grown to comparable thicknesses in the range of 20–25 nm. Evaluation of their mechanical behavior indicated that the properties could be approximated to those of sequentially stacked hard and soft layers, and vice versa, on monocrystalline silicon. Both hardness and elasticity of the double layers essentially depended on the order of constituent oxide films of different durability. The ZrO_2_/SnO_2_/Si stacks with ZrO_2_ top layers could be characterized with a hardness of 11 and elastic modulus of 72 GPa, whereas in the SnO_2_/ZrO_2_/Si stacks, the corresponding values reached 15 and 171 GPa.

The double-layered nanostructures exhibited magnetization behavior characteristic of ferro-, ferri-, or paramagnetic materials, i.e., nonlinear and reaching saturation in external magnetic fields. The ZrO_2_/SnO_2_/Si stacks possessed higher magnetization values compared to those in the SnO_2_/ZrO_2_/Si stacks, but the latter films demonstrated stronger coercive force, 130 versus 65 Oe. Such studies may add to the knowledge on the synthesis and characterization of wide-band-gap, and thereby optically transparent, metal oxide-based composites as mechanically tough and, at the same time, magnetically susceptible thin films.

## Figures and Tables

**Table 1:** Thickness, density, and roughness values of representative nanolayered thin films obtained by X-ray reflection measurements.

	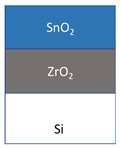	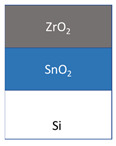	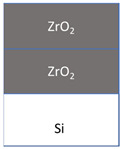	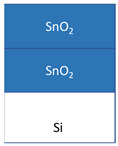
Description of the Given Layered Nanostructures	SnO_2_/ZrO_2_/Si	ZrO_2_/SnO_2_/Si	ZrO_2_/Si	SnO_2_/Si
Thickness, nm	14.9 (4)/10.4 (3) Total 25.3 nm	11.4 (2)/10.1 (9) Total 21.5 nm	21.0 (2)	24.9 (2)
Density, g/cm^3^	7.02/5.82	5.68/6.80	5.68	6.95
Roughness, nm	1.2 (4)	1.7 (2)	2.1 (1)	1.4 (8)

**Table 2:** Descriptive statistics of nanoindentation results (SD standard deviation).

Sample	Hardness (GPa) Geometric Mean ± SD	Hardness (GPa) Range	Young’s Modulus (GPa) Geometric Mean ± SD	Young’s Modulus (GPa) Range
ZrO_2_/Si	11.5 ± 1.1	7.7–13.5	96 ± 1	84–119
SnO_2_/Si	14.8 ± 1.1	12.0–18.8	175 ± 1	142–229
ZrO_2_/SnO_2_/Si	10.8 ± 1.1	7.5–15.3	72 ± 1	62–89
SnO_2_/ZrO_2_/Si	15.1 ± 1.1	11.6–20.0	171 ± 1	143–205
Si	10.8 ± 1.2	7.5–14.0	133 ± 1	108–167

## Data Availability

Not applicable.
